# How do family physicians communicate about cardiovascular risk? Frequencies and determinants of different communication formats

**DOI:** 10.1186/1471-2296-12-15

**Published:** 2011-04-05

**Authors:** Stefan Neuner-Jehle, Oliver Senn, Odette Wegwarth, Thomas Rosemann, Johann Steurer

**Affiliations:** 1 Institute of General Medicine and Health Services Research, University of Zürich, Switzerland; 2 Max Planck Institute for Human Development, Harding Center for Risk Literacy, 14195 Berlin, Germany; 3 Horten-Center for patient oriented research and knowledge transfer, University of Zürich, Switzerland

**Keywords:** risk communication, risk formats, cardiovascular risk factors, cardiovascular disease, primary care

## Abstract

**Background:**

Patients understand information about risk better if it is communicated in numerical or visual formats (e.g. graphs) compared to verbal qualifiers only. How frequently different communication formats are used in clinical primary care settings is unknown.

**Methods:**

We collected socioeconomic and patient understanding data using questionnaires and audio-recorded consultations about cardiovascular disease risk. The frequencies of the communication formats were calculated and multivariate regression analysis of associations between communication formats, patient and general practitioner characteristics, and patient subjective understanding was performed.

**Results:**

In 73% of 70 consultations, verbal qualifiers were used exclusively to communicate cardiovascular risk, compared to numerical (11%) and visual (16%) formats. Female GPs and female patient's gender were significantly associated with a higher use of verbal formats compared to visual formats (*p *= 0.001 and *p *= 0.039, respectively). Patient subjective understanding was significantly higher in visual counseling compared to verbal counseling (*p *= 0.001).

**Conclusions:**

Verbal qualifiers are the most often used communication format, though recommendations favor numerical and visual formats, with visual formats resulting in better understanding than others. Also, gender is associated with the choice of communication format. Barriers against numerical and visual communication formats among GPs and patients should be studied, including gender aspects. Adequate risk communication should be integrated into physicians' education.

## Background

A major challenge in preventive medicine is to effectively inform patients about the risks and benefits of possible treatment options. Risk can be communicated by words (verbal qualifiers, e.g., "your risk is high" or "this is not good for your health"), by numerical formats (absolute percentages, relative percentages, natural frequencies), by visual formats, or by a combination of these methods [1,2]. Although there is no clear evidence as to which format is the best for communicating risk most effectively, recent research allows a ranking (hierarchy) of the different risk communication formats in terms of effectiveness and patient understanding: the use of natural frequencies, graphical formats (e.g., bar charts), and combinations of these are more comprehensive than percentages or a purely verbal translation of risk [3-6], and patients prefer these formats as opposed to percentages [7]. Patient characteristics as age, education, cultural background, psychosocial aspects as well as literacy influence a patient's understanding of information about risk and communication preferences [8]. The numeracy of patients refers not only to the capacity of understanding numerical formats accurately, but also to graphical formats. Numeracy has an impact on risk perception, adherence to interventions and even health outcomes [9]. Also physician characteristics are important determinants of which formats are used, thus leading to a large variation in the use of risk communication formats among primary care providers [10]. Growing evidence suggests that involving patients in decision-making has positive effects in terms of patient satisfaction, adherence, and even health outcomes [11,12]. Patients increasingly seek more active participation in healthcare decisions, though not all of them to the same degree [13], and there has been a call for a shift towards a meaningful dialogue between patients and physicians and shared decision making [14].

Cardiovascular disease (CVD) is a major issue in public health, contributing excessively to the overall morbidity and mortality of populations in industrialized societies [15]. Only a minority of people with cardiovascular risk factors (CVRF) who qualify for prophylactic interventions to lower their risk is treated adequately [16,17]. Possible explanations are low adherence of doctors to guidelines and ineffective risk communication between doctors and patients [18]. Furthermore, cardiovascular risk is often perceived inappropriately by primary care patients, leading to over- or underestimation of the risk. Communicating risk by understandable formats has the potential to correct inappropriate risk perception [19]. Systematic reviews suggest that providing adults at moderate to high CVD risk with information about their global CVD risk (using different communication formats) improves their accuracy of risk perception and probably increases their intent to initiate CVD prevention [12,20]. Gain-framed messages (e.g. presenting a survival benefit), as apposed to lost-framed messages (e.g. presenting a potential damage), shorter timeframes (5 or 10 years until realisation of the risk, versus 15 or 20 years) and visual formats seem to enhance understanding of risk and to increase the self-efficacy to prevent CVD [21].

Thus, improving comprehension and the effectiveness of risk communication in the field of CVD is an important and challenging task in medicine with public health consequences. How often primary care physicians use the various risk communication formats while counseling patients with CVRF is yet unknown.

The aim of this study was to investigate how frequent verbal, numerical, and visual formats, or a combination of formats, are used in the CVRF communication process between primary care physicians and patients. An additional aim was to identify patient and GP characteristics associated with different counseling formats. In contrast to previous research studying the effectiveness of different risk communication formats in healthy volunteers [2,4,6], we chose a real physician-patient encounter and assessed the primary outcome (counseling format) by audio-recordings rather than self-reporting.

## Methods

### Procedure

To investigate how CVD risk is communicated, primary care physicians audio-taped counseling sessions with patients at CVD risk. The sessions were part of the usual care performance of the general practitioners (GPs; specialists for general and internal medicine). In three northern and central Swiss cantons (Zurich, Lucerne, and Zug), all GPs approved by the Swiss Medical Doctors Federation (Foederatio Medicorum Helveticorum, FMH) who were either running a medical office independently or employed in a medical office were invited to participate by information leaflet sent to postal addresses provided by the FMH. The recruitment and instruction of doctors started in December 2008, and data were collected until the end of February 2010. The study was received and approved by the ethical committees of the cantons of Zurich, Lucerne and Zug.

The doctors who were willing to participate were visited and provided with information about the aims of the study in so far that we told them that the study is about investigating risk communication in daily practice. The participants were further introduced to the details of how to collect the requested data and provided with the required materials (digital audio-recorder, several copies of a GP questionnaire, several copies of a patient questionnaire), as they had to collect all data by themselves. No suggestions were given to physicians on the method or program of risk calculation. No information was given neither to physicians nor patients as to what we exactly planned to analyze in the interviews, in order to avoid desirability bias. The doctors were informed about the planned analysis only after data collection, including the right to withdraw the data (which none of the GPs finally did).

During the next three months, GPs identified eligible patients among those in their practice and asked them to participate based on the following inclusion criteria: 35 to 65 years of age (in order to adapt to the age range of risk calculators; exceptionally younger or older participants could be included if the GP calculated their risk by other methods), and with at least one risk factor of the following - lipid disorder; high blood pressure (using internationally accepted cut-off points), and ongoing tobacco smoking. Exclusion criteria were: cognitive deficiency (dementia, stroke); terminal disease with poor prognosis in terms of survival time; acute somatic or psychiatric disorder; and clinical manifestation of any CVD. Patients willing to participate signed an informed consent form before starting the counseling session. These sessions were audio-taped, and patients and GPs completed a questionnaire immediately after the session.

Patient questionnaires referred to their age, gender, level of education, ethnicity, and the presence of CVD in first degree relatives. The self-rated understanding of the received information, the awareness (estimate) of the risk of developing CVD, and the anxiety regards developing CVD were measured on a visual analogue scale (VAS) ranging from 0 (low level of understanding, awareness, and anxiety) to 100 (highest level of comprehension, awareness, and anxiety) points. In addition, patients documented a translated 8-item-set of a validated index of the need and expectation for medical information, the autonomy preference index (API) [22,23]. The GP questionnaire asked for an estimate of the patient's risk of developing CVD and an estimate of the patient's anxiety towards developing CVD, again measured on a VAS, ranging from 0 (low level of risk and anxiety) to 100 (highest level of risk and anxiety) points.

All audio tapes and questionnaires were collected by the principal investigator three months after the first visit. After the recorded files were transcribed, data from each counseling session were classified independently by two researchers into different format categories: verbal, numerical, visual, or combined. In case of disagreement, discussion of the interview would follow in order to reach consensus; if this would not be possible, data would be excluded. In fact, there was no disagreement among raters in the communication format classification of interviews.

A consultation was classified as „visual format" if the information from the audio-taped interview indicated the use of a table or graph. The total counseling time and the ratio of patient talking time to total consultation time was calculated from the audio record.

### Participants

Out of 1188 primary care physicians invited to participate in the study, 35 (2.9%) were willing to participate. Finally, 22 primary care physicians (1.9% of invited GPs) enrolled 77 patients (1-9 patients each). For seven patients, audio records were incomplete due to technical reasons and, therefore, excluded from the main analysis. No values were missing in the questionnaires. The baseline characteristics of the physicians and patients are summarized in Table 1. The median age of the participating primary care physicians was 46.5 years (IQR 38 - 57 years), and 64% were male. The GPs had a median 11 years (IQR 5 - 20 years) of experience as a primary care physician. The median age of patients was 51 years (IQR 45.5 - 59 years), and 58% were male.

**Table 1:** Baseline characteristics of physicians (n = 22) and patients (n = 77)

Physician characteristics	Median	IQR	n	% of cases
Age, years	46.5	38-57		
Experience as a GP, years	11	5-20		
Workload, % (100% = 5 days working per week)	100	77.5-100		
Sex, males			14	63.6
Practice type, solo			9	40.9
Practice location, urban			10	45.5
rural			12	54.5
				
**Patient characteristics**				
Age, years	51	45.5-59		
Sex, male			45	58.4
Ethnicity, Swiss origin			66	86.8
Education level, Primary			11	14.3
Secondary			38	49.4
High school			5	6.5
Academic			23	29.9
Number of cardiovascular risk factors = 1			49	63.3
2			23	29.9
3			5	6.5
Cardiovascular risk factor				
Lipid disorder			41	53.2
Hypertension			45	58.5
Tobacco smoking			24	31.2

Total			110	142.9

### Data analysis

For our main outcome, the prevalence of different risk communication formats, we calculated the frequency of each format and the corresponding 95% confidence intervals. We tested bivariate associations between the different counseling formats and patient and physician characteristics using the Kruskal-Wallis and Fisher's exact tests for continuous and categorical variables, respectively. The intraclass correlation coefficient was calculated to assess the agreement between different counseling formats in GPs who recorded at least two encounters. To further investigate independent determinants of the counseling format used (verbal-numerical-visual) we performed a multinomial logistic regression analysis including all physician and patient characteristics showing at least a borderline significant (i.e. *p*<0.1) bivariate association with the counseling format. In addition, the model was controlled for the clustering effect by the GP. In a secondary analysis, we investigated whether the counseling format was an independent determinant of the level of understanding by performing a multiple linear regression. Potential confounding factors, such as patient age and gender, education, API, total counseling time, patient's self-assessed level of CVD risk, and anxiety, were included as covariates. For the quantitative analysis, we used the SPSS Statistical Software Package (SPSS version 14, SPSS Inc, Chicago, Illinois).

## Results

In 51 of 70 consultations (73%; 95% CI 62-84%), GPs communicated cardiovascular risk to their patients using verbal qualifiers only. In eight consultations (11%; 95% CI 4-19%), the GPs combined such verbal qualifiers with numerical information. Graphical formats were exclusively used in one consultation (1.4%; 95% CI 0.3-8%) and together with numerical information in 10 consultations (14%; 95% CI 8-24%).

Among the 18 consultations in which GPs presented *numerical *information to their patients, they exclusively used absolute percentages in 10 sessions, combined absolute risk information and natural frequencies in seven sessions, and used relative risk information to communicate the patient's risk in one session.

The majority of *visual formats *(91%) were tables with a color-coding system analogous to traffic light colors, indicating low, medium, and high risk values. The underlying risk calculation was always based on a 10-years timeframe. Only one doctor used another graphical format, a survival curve. Bar charts or population diagrams were not used at all in our study sample.

Ten of 16 GPs (62.5%) who provided more than one consultation used the same risk communication format throughout the sessions without variation, whereas six GPs switched between different formats. The intraclass correlation for the counseling formats used was 0.63 (95% CI 0.41-0.85; *p < 0.01*) in GPs who performed more than one consultation on risk, indicating a significant clustering effect.

The mean consultation time for communicating risk was 9 min 46 sec (SD, 5 min 35 sec), ranging from 1 min 26 sec to 32 min 04 sec. During the consultations, patients were talking 24% of the time. On average, GPs' estimate of patients' CVD risk was 29 (range: 0-81) on the VAS scale (0-100), whereas patients' mean estimate of their own CVD risk was 28 (range: 3-100). In the direct comparison between GPs' and patients' CVD risk estimates (estimates on the same patient), they differed by 17.5 points (SD 17.3). For the estimates of the anxiety of developing CVD, the mean estimate of GPs was 31 (range: 1-98), the mean estimate of patients 26 (range: 0-100). On average, the estimates of GPs and patients differed by 16.0 points (SD 16.4) in the direct comparison.

**Figure 1:**
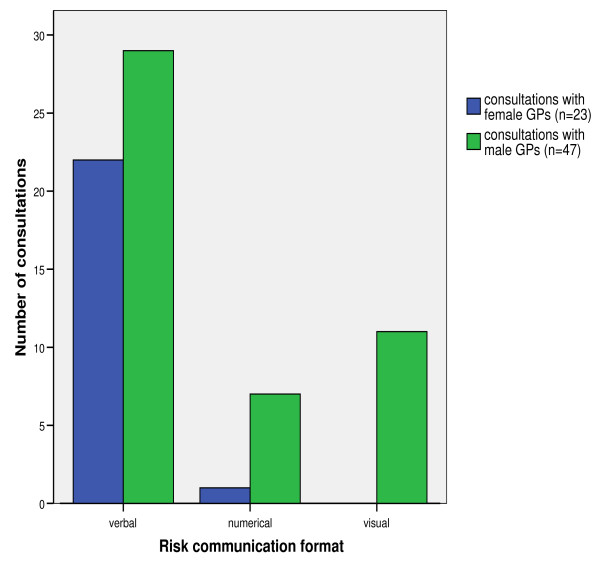
**Applied risk communication format depending on the gender of the GPs (bars show the frequencies of consultations conducted by female GPs and male GPs)**.

**Figure 2:**
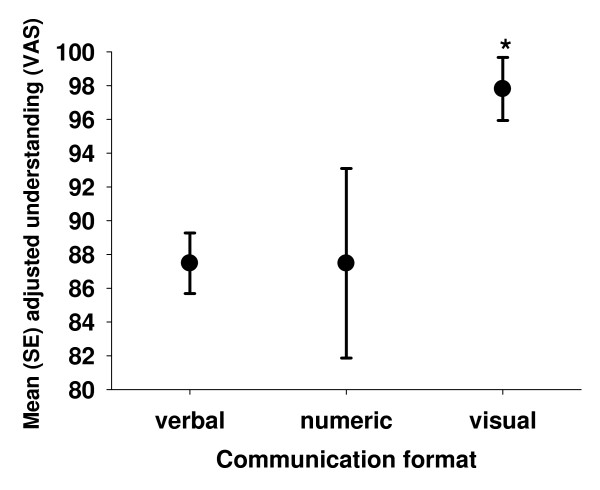
**Association between the subjectively perceived level of understanding and the applied risk communication format**. Difference between the communication formats regarding to the mean estimates of subjectively perceived unterstanding: *p *= 0.001 for visual vs. verbal format and *p *= 0.12 for visual vs. numerical format.

Results of the bivariate analysis between risk counseling formats and patient and physician characteristics are listed in Table 2. *Gender *was the only GP characteristic strongly associated with the communication format; only one female physician used numerical formats and none used visual formats (Figure 1). Patient gender and degree of subjective understanding the given information were patient determinants associated with the communication format. Female patient gender remained significantly associated with a higher use of purely verbal qualifiers compared to visual formats when controlled for practice type (i.e. a single workplace medical office vs. a primary care center with several physicians), duration of GP-patient relationship, patient subjective understanding, the ratio of patient talking time to total consultation time, and the clustering effect of the GP (OR 1.4, *p *= 0.039). The use of a visual format resulted in significantly higher subjective perceived patient understanding compared to pure verbal counseling (adjusted mean (SE) difference of 10.3 (2.7) points on the 0-100 VAS scale), which remained independently associated when controlled for patient age and gender, education, API, total counseling time, patient's self-assessed level of CVD risk and anxiety, and GP clustering effect (Figure 2). None of the other potential covariates analyzed showed any association with the communication format.

**Table 2:** Patient and GP characteristics and communication formats

Characteristic		Communication format			*p*
		verbal	numeric	visual	
Practice location	Urban	15 (21.4)	4 (5.7)	6 (8.6)	0.196
	Rural	36 (51.4)	4 (5.7)	5 (7.1)	

Practice type	Solo	17 (24.3)	1 (1.4)	7 (10.0)	0.080
	Multiple	34 (48.6)	7 (10.0)	4 (5.7)	

Experience as GP (years)		10 (5.5-19.5)	13 (12.5-21.5)	13.5 (8-19)	0.149

GP age (years)		46 (38-56)	59 (52.5-59)	49.5 (44-55)	0.470

Workload (%)		80 (60-100)	100 (78-100)	100 (100-100)	0.277

GP gender	Female	22 (31.4)	1 (1.4)	0*	0.004
	Male	29 (41.4)	7 (10.0)	11 (15.7)	

Duration of relationship between GP and patient		5 (2-8)	5 (3.5-12.5)	1.5 (1-5)	0.067

Patient gender	Female	26 (37.1)	1 (1.4)	2 (2.9)	0.035
	Male	25 (35.7)	7 (10.0)	9^# ^(12.9)	

Patient age, years		51 (45.5-59)	55 (55-58.5)	53.5 (49-58)	0.610

Patient ethnicity	Inborn	45 (65.2)	8 (11.6)	8 (11.6)	0.152

	Foreign	5 (7.2)	0	3 (4.3)	

Patient education level					
	Primary school	9 (12.9)	0	0	0.372
	Secondary school	27 (38.6)	4 (5.7)	5 (7.1)	
	High school/university	15 (21.4)	4 (5.7)	6 (8.6)	

Number of cardiovascular RF	1	33 (47.1)	5 (7.1)	7 (10.0)	0.372
	2	16 (22.9)	2 (2.9)	2 (2.9)	
	3	2 (2.9)	1 (1.4)	2 (2.9)	

Cardiovascular event in family	Yes	22 (31.4)	2 (2.9)	4 (5.7)	0.680
	No	29 (41.4)	6 (8.6)	7 (10.0)	

Estimation on CVD risk by GP (points on a 0-100 VAS)		25 (14.5-50.5)	25 (20-29)	30 (22-52)	0.354

Estimation on CVD risk by patient (points on a 0-100 VAS)		28 (18.5-46.5)	26 (21.5-39)	38 (18-50)	0.760

Estimation of anxiety by GP (points on a 0-100 VAS)		31 (14-49.5)	27 (23-48)	19.5 (10-31)	0.292

Estimation of anxiety by patient (points on a 0-100 VAS)		28 (13.5-50.5)	15 (9.5-42)	19.5 (3-47)	0.242

Need for information (points on the 1-5 API scale)		1.125 (1-1.375)	1.375 (1.25-1.438)	1.25 (1.125-1.675)	0.252

Patient's comprehensione of the given information (points on a 0-100 VAS)		94 (86-97.5)	96 (73-97.5)	99 (96-100)^#^	0.026

Total consultation time (min)		9 (6-12)	10 (7-15)	10 (8.6-12)	0.70

Ratio of patient talking time to total consultation time (%)		23 (16-24)	14 (12-18)	19 (12-24)	0.070

Data are given as median (IQR) or n(%) and do not necessarily sum to n = 77 due to missing values.The Kruskal Wallis test for continuous data and Fisher's exact test for categorical data were performed to test for overall effect across counseling formats.

* *p*<0.05 vs. verbal, # *p*<0.05 vs. numerical and vs. verbal.

## Discussion

The majority (73%) of primary care physicians participating in this study used exclusively verbal formats to communicate cardiovascular risk to their patients. The combination of numerical and visual formats was used to a minor degree, as was the combination of verbal and numerical formats. Our findings demonstrate a gap between the recommendations of medical associations, which favor numerical and visual formats for communicating risk, and the reality in clinical practice.

In our study, the frequency of the use of verbal formats in risk communication appeared to be associated with gender; female physicians communicated risk more often in verbal formats than male physicians. We found the same association, though to a lesser degree, in female patients, independent of the GP's gender. There are only few data existing on this gender issue in doctor-patient communication; a recent systematic review showed that female doctor-patient dyads are talking longer and combine different communication styles in one consultation in comparison to mixed or male dyads [24]. To our knowledge, the gender association in risk communication formats has not been described previously and merits further research. This tendency of female physicians and female patients to communicate exclusively in a verbal format should also be addressed in the development of medical education programs and tools.

The statistically quantifiable association between the use of visual formats and understanding rated by patients in our study is not necessarily translating into a clinically relevant benefit of understanding among patients. Though, it is consistent with data from the literature, which show that visual formats are easier to understand than other, especially verbal, formats [5-7]. Only a few studies address the visual formats used by the physicians in our study [25]. However, compared to verbal and numerical formats alone, findings for other visual formats such as "risk ladders" [26], population charts [27], pie charts [28], and histograms [29-31] suggest better understanding, increased risk perception, higher risk aversion, and better acceptance of interventions in patients. Restrictions on the use of visual formats should not be forgotten: depending on the skills of a person to understand graphics [32] and the danger to manipulate patients by emphasizing parts of the graphic [33-36]. The same is true for numerical formats. Moreover, some visual formats (e.g., survival curves and scales) need additional verbal or numerical explanations to be sufficiently clear [37,38], and others, such as population figures, are not easier to understand than numerical formats [39]. The use of colors as additional information in visual formats, which was also used by participating physicians, seems to be powerful and familiar to patients, providing them with an important reference point about the severity of risk (e.g., the red color indicating urgency and necessity to stop proceeding in the same way as until now) [40].

Nevertheless, a high proportion of patients in our sample indicated that they subjectively understood the communicated risk fairly well when confronted with verbal qualifiers or numerical formats alone. Sixty-three percent of GPs used their preferred format without variation, which indicates that, in addition to the associations with doctor and patient gender, the choice of the communication format depends more on physician characteristics than those of the patient.

The difference between GPs' and patients' estimates of CVD risk and the anxiety of developing CVD is significant. Previous research already reported a high frequency of underestimation of CVD risk (and, to a minor degree, overestimation) among patients with CVD risk [19]. In the absence of biometric data from patients in our study population in order to calculate their CVD risk, it is not possible to tell whether rather patients' or GPs' estimate of risk is closer to the real CVD risks. The result is nevertheless noteworthy as it makes obvious that to a certain extent physicians are not able to fully transfer their risk estimation to their patients.

### Strengths

This study is the first to provide data from a real clinical setting targeting the use of different formats in communication of CVRF between physicians and patients. We chose a study design that optimally handles desirability or reporting bias, in contrast to studies using self-reported data from questionnaires, as our main covariate (i.e. risk communication format) is not self-reported but recorded and objectively analyzed.

### Limitations

Due to the small sample size, especially the number of GPs, the generalizability of our results is limited. The participation rate of doctors (1.9%) was low. We think that GPs are not used to making audio recordings of their own consultations, and this may be a major psychological barrier for participation. The drop-out rate of 37% was explained by the physicians who were willing to participate but unable to enroll patients because of their high work load and lack of time. Physician age, gender, and experience in ambulatory primary care in our sample matches well with the statistics reported by the FMH, but the sample size is far too small to be representative of the entire population of Swiss primary care doctors. Moreover, the recruitment strategy might have led to selection bias towards a group of doctors with more motivation and skills in communication techniques. However, this bias may support our findings as one would expect that less motivated colleagues might also be less likely to catch up with the recent recommendations of their medical associations.

### Possible explanations of the findings

One reason for the high extent of verbal qualifier use compared to other formats might be that primary care physicians are not sure about the numerical facts regarding risk. Several studies suggest that, regardless of specialization, physicians often face problems understanding medical statistics [41-43]. Also, many of the physicians may not know the effects of different formats of risk communication on patient understanding and their emotional perception of risk. Many physicians might judge risk presentation as verbal "value" to be good enough, not being aware of the low correlation between objective risks and perceived risk in patients. Similarly, the need and desire of patients to obtain maximum information, which was also shown in our study by the API, might be underestimated by physicians.

A second reason is that teaching activities regarding communication skills are still at a low level in the professional education of Swiss physicians. Consequently, many doctors do not feel comfortable with different communication techniques. Furthermore, analogous to findings in the use of calculation and communication tools to predict cardiovascular risk in primary care, doctors face many barriers, such as distrust in their validity or their contribution to encourage decision making, lack of time, and low reimbursement [44].

Another issue contributing to our observations is the *trust and confidence *of patients in their doctors. As we know from research in the field of shared decision making, many patients prefer not to decide themselves, but to leave the decision to the doctor [13], or prefer to know his opinion rather than being informed of facts [11]. Asked for a *personal opinion*, the physician may prefer verbal communication formats to others, taking the risk of inducing different meanings and confusion in patients. Similarly, by purposes like reassuring or persuading, GPs may prefer verbal formats, as was shown e.g. for clinical geneticists [45]. The asymmetry in mean talking time indicates a more one-sided sort of communication (with the physician as a source of information), rather than an interactive discussion among equal partners. Moreover, the straightforward use of verbal qualifiers by doctors is a time-saving approach not requiring any preparation time, unlike choosing adequate numbers or drawing graphs.

At present, several programs are in development in Switzerland, yielding to patient-centered health-promoting activities in primary care settings [46]. Within these programs, risk communication is a major issue. Visual formats like colour-coded graphics are emphasized, in order to transport information about risk to patients in the most understandable way and to facilitate discussion about risk between physician and patient [47].

## Conclusion

In summary, our data demonstrate a gap between the recommendations from medical associations and clinical reality in communicating CVD risk. The verbal formats that are mainly used are rated lowest in recommendations regarding understanding and effectiveness. Similarly, the highly recommended formats, such as natural frequencies and visual formats like bar charts, were rarely or never used by the primary care physicians in our study. Visual formats resulted in significantly higher subjective understanding of the information given. Also, gender is significantly associated with the choice of communication formats, which was unknown thus far for CVD risk communication.

As implication for clinical practice, relevant barriers towards the use of "highly ranked" communication formats among doctors and patients are to be identified and addressed. Furthermore, strategies for improving communication skills among doctors should be developed, such as practicable tools and medical education programs. The gender aspect should be addressed, especially in regard to target groups of interventions. The results of our study stress the need for developing health-promoting programs in primary care that would more clearly focus on a transparent communication on risk. Our final intention is to close the gap between theory and daily clinical practice in the field of CVRF communication, which does not necessarily mean that doctors and patients have to change their communication style - it might lead to input for the further development of theoretical models adapted to reality.

## List of abbreviations

CVD: cardiovascular disease; CVRF: cardiovascular risk factor; VAS: visual analogue scale; FMH: Swiss federation of physicians; GP: general practitioner; IQR: interquartile range; API: autonomy preference index; SD: standard deviation.

## Competing interests

The authors declare that they have no competing interests.

## Authors' contributions

SNJ conceived the study, carried out data collection, performed statistical analysis and drafted the manuscript. OW participated in the design and writing of the manuscript. OS participated in statistical analysis and writing of the manuscript. JS and TR participated in the design of the study. All authors read and approved the final manuscript.

## Pre-publication history

The pre-publication history for this paper can be accessed here:

http://www.biomedcentral.com/1471-2296/12/15/prepub
